# The implication of cardio-oncology on Parkinson's disease: answers begin to emerge

**DOI:** 10.1016/j.clinsp.2022.100085

**Published:** 2022-08-03

**Authors:** Fulvio A. Scorza, Antonio-Carlos G. de Almeida, Josef Finsterer, Ludhmila A. Hajjar

**Affiliations:** a Disciplina de Neurociência, Escola Paulista de Medicina, Universidade Federal de São Paulo (UNIFESP), São Paulo, SP, Brazil; b Centro de Neurociências e Saúde da Mulher “Professor Geraldo Rodrigues de Lima”, Escola Paulista de Medicina/Universidade Federal de São Paulo (EPM/UNIFESP), São Paulo, SP, Brazil; c Laboratório de Neurociência Experimental e Computacional, Departamento de Engenharia de Biossistemas, Universidade Federal de São João del-Rei (UFSJ), São João del-Rei, MG, Brazil; d Klinikum Landstrasse, Messerli Institute, Vienna, Austria; e Instituto do Coração, Faculdade de Medicina da Universidade de São Paulo (FMUSP), São Paulo, SP, Brazil; f Instituto do Câncer, Universidade de São Paulo (USP), São Paulo, SP, Brazil

Parkinson's Disease (PD) is a progressive, incurable, and symptomatically devastating neurodegenerative disease.^1^, ^2^, ^3^ PD is the second most common neurodegenerative disease affecting up to ten million people worldwide.^4^ Clinically, PD presents with tremor, rigidity, bradykinesia/akinesia, and postural instability due to the progressive loss of neurons in the Substantia Nigra pars compacta *(*SNpc) and extensive intracellular aggregation of α-synuclein.^3^, ^4^, ^5^ In addition, molecular mechanisms such as mitochondrial dysfunction, excitotoxicity, impaired autophagy, oxidative stress, and genetic mutations are implicated in the degeneration of dopaminergic neurons in PD.^6^ Available therapies for PD only affect the symptoms of the disease and mainly focus on the dopaminergic pathway.^2^^,^^3^^,^^5^^,^^7^ The most common non-motor symptoms associated with PD involve neurobehavioral complications, sensory problems, and autonomic dysfunction.^6^ Several studies have found autonomic dysfunction to be a valuable predictor of cardiovascular morbidity and mortality in PD patients.^8^^,^^9^ Despite the tremendous clinical and epidemiological relevance of these studies, Sudden Death in PD (SUDPAR), a rare but fatal event, contributes to the increased mortality in PD patients.^2^^,^^10^

Although the authors experienced an enlightening phase in PD research in the last decade,^2^^,^^3^ data on the influence of comorbidities on morbidity and mortality in PD are still lacking. Previous studies showed that hypertension, back problems, arthritis, dementia, cataracts, diabetes *mellitus* type II, non-specific hyperlipidemia, depressive disorder, atrial fibrillation, and urinary incontinence were the most common comorbidities in PD patients.^11^, ^12^, ^13^ There is also growing evidence that PD and cancer may be closely linked.^14^^,^^15^ In fact, cancer incidence increases with age, as does the incidence of PD.^14^^,^^16^ On the one hand, an increased incidence of melanoma and other skin tumors, brain cancer, thyroid, and breast cancer has been demonstrated in PD patients.^6^^,^^17^ On the other hand, other studies showed that PD is associated with a significantly reduced risk of lung cancer, genitourinary cancers, gastrointestinal cancers, and hematological cancers.^6^^,^^17^ Although these data are controversial, there is a putative link between PD and cancer that requires much caution and further studies.^6^^,^^17^ Based on all these data and the worrying situation, the authors think it is appropriate to relate PD to the multidisciplinary specialty known as cardio-oncology.^18^ In particular, the main goal of cardio-oncology is the prevention and treatment of cancer therapy-related cardiotoxicity to optimize cardiovascular health outcomes in cancer patients or after cancer.^18^^,^^19^ In fact, it is already recognized that some cancer treatments can lead to atrial tachyarrhythmias, ventricular arrhythmias, and bradyarrhythmias.^19^ Therefore, based on the authors’ experience of more than a decade with the cardio-oncology program in Brazil,^20^ it is necessary to apply this knowledge to patients with PD as well. Certainly, the authors fully agree^18^ that this valuable clinical convergence will enable us to identify PD patients at risk for cardiac complications and to develop precise cardioprotective strategies.

What lessons have the authors learned overall in recent years? Firstly, with an aging population and rising life expectancy worldwide, PD is estimated to increase by more than 50% by 2030.^2^^,^^7^ Second, PD is a systemic disease with cases of premature death. SUDPAR is a rare event, but it is common in PD patients with positive family history and risk factors for cardiovascular disease. Third, several studies suggest that cancer may be associated with PD. Furthermore, as extraordinary advances in translational neuroscience have changed our understanding of PD in the last decade,^2^ it is extremely important to integrate the concept of cardio-oncology in centers for movement disorders. In fact, as cancer survival has increased over time due to improvements in treatment, it is reasonable to expect that the significant risk of cardiovascular complications for survivors will remain high in the near future.^21^ In these lines, the authors are in agreement that new lines of research should converge the research expertise of not only cardiologists and oncologists, but potentially others, including neurologists, and those with translational research experience.^21^ Finally, all medical specialties must work together in research, education, and collaboration to ensure healthy lives of the patients in cardio-oncology.

## Conflicts of interest

The authors declare no conflicts of interest.
